# Lady Pharmacists

**Published:** 1881-12-20

**Authors:** 


*Lady Pharmacists in Holland.*—Notwithstanding the gallantry of the medical laws of Holland, lady students of pharmacy in that country have hitherto contented themselves with the diploma of pharmaceutical assistant. One of them, Mlle. Jacobs, has, however, lately conquered the higher grade of pharmacien.—*J. de Pharm d'Anvers.*

